# Clinical and Laboratory Parameters of Carp Edema Virus Disease: A Case Report

**DOI:** 10.3390/v15051044

**Published:** 2023-04-25

**Authors:** Ivana Papežíková, Veronika Piačková, Iva Dyková, Ali Asghar Baloch, Hana Kocour Kroupová, Eliška Zusková, Ľubomír Pojezdal, Hana Minářová, Eva Syrová, Hana Banďouchová, Pavel Hyršl, Kateřina Matějíčková, Jiří Pikula, Miroslava Palíková

**Affiliations:** 1 Department of Zoology, Fisheries, Hydrobiology and Apiculture, Mendel University in Brno, 613 00 Brno, Czech Republic; 2 Department of Ecology and Diseases of Zoo Animals, Game, Fish and Bees, Faculty of Veterinary Hygiene and Ecology, University of Veterinary Sciences Brno, Palackého tř. 1946/1, 612 42 Brno, Czech Republic; 3 University of South Bohemia in České Budějovice, Faculty of Fisheries and Protection of Waters, South Bohemian Research Centre of Aquaculture and Biodiversity of Hydrocenoses, Zátiší 728/II, 389 25 Vodňany, Czech Republic; 4 Department of Botany and Zoology, Faculty of Science, Masaryk University Brno, 625 00 Brno, Czech Republic; 5 Department of Infectious Diseases and Preventive Medicine, Veterinary Research Institute Brno, 621 00 Brno-Medlánky, Czech Republic; 6 Department of Experimental Biology, Masaryk University, 625 00 Brno, Czech Republic

**Keywords:** koi sleepy disease, immunology, respiratory burst, histology

## Abstract

In the present study, we describe a natural outbreak of carp edema virus disease (CEVD) in koi carp, concentrating on clinical manifestation, gross and microscopic pathology, immunological parameters, viral diagnostics, and phylogenetic analysis. Examination of white blood cell parameters showed increased monocyte and decreased lymphocyte counts in CEV-affected fish compared to healthy control fish. Regarding immune system functioning, the present work shows, for the first time, enhanced phagocytic activity in CEV-affected fish. Respiratory burst of phagocytes was strongly increased in diseased fish, the increase being attributed to an increased phagocyte count rather than enhancement of their metabolic activity. The present work also newly shows histopathological changes in the pancreatic tissue of diseased koi.

## 1. Introduction

Carp edema virus disease (CEVD), also known as koi sleepy disease (KSD), is an emerging threat to koi and common carp (*Cyprinus carpio*) aquaculture. CEVD was first reported in Japan in 1974, where it caused heavy mortalities and high economic losses in koi carp aquaculture [1,2]. Carp edema virus (CEV) has now spread globally, with clinical cases having been reported from both North and South America [3,4], Asia [5,6,7], and numerous European countries. In Europe, CEVD was first reported in the UK by Way and Stone [8], followed by reports of further outbreaks in the Netherlands [9], Czech Republic [10], Austria [11], Italy [12], Poland [13], France [14], Germany [15], and Slovakia [16].

CEV is a double-stranded DNA virus of the *Poxviridae* family [11]. The gills are the primary target organ and the primary source of virus identification in diseased fish [1,17,18]. Affected carp show behavioural abnormalities, including lethargy and unresponsiveness (hence the alternative name of the infection, ‘koi sleepy disease’), with fish often seen lying at the bottom of a tank for long periods of time as if sleeping [18]. External clinical signs include haemorrhagic skin lesions, generalised edema, pale and swollen gills, sunken eyes (enophthalmia), and anal ulcerative inflammation [9,11,13]. In some cases, mucus overproduction on the skin and gills may also be observed [12,19]. Mortality can reach up to 100% [20].

Measurement of immunological parameters is very useful to evaluate immune responses to different stressors, including infectious disease agents. To increase our understanding of CEVD pathophysiology, we examined immunological parameters alongside examination for clinical manifestations, gross and microscopic pathology, viral diagnosis, and phylogenetic analyses.

## 2. Materials and Methods

### 2.1. Case description and Fish Sampling

At the beginning of April 2017, increased mortality and unusual behaviour (extreme lethargy, unresponsiveness and lying at the bottom of the tank) were observed in koi carp kept by a private vendor of ornamental fish. The fish were imported from Japan in mid-February 2017 and, after one month of quarantine, were translocated to the owner’s facility, where clinical symptoms started 2–3 weeks later. The water temperature at the time of occurrence of first clinical symptoms was 12 °C. Total mortality reported by the owner was estimated at 50%.

On 4 April 2017, two live fish were delivered for examination (Sampling A). As PCR examination of samples from Sampling A were positive for CEV (see below), further sampling was performed (Sampling B) on 17 April 2017, during which ten fish showed severe clinical signs (mean total length 134.2 ± 12.12 mm; mean body weight 30.2 ± 10.39 g) and ten control fish showing no symptoms (mean total length 159.1 ± 27.64 mm; mean body weight 59.30 ± 31.26 g) were collected. The control fish were collected from a different tank with a separate water circulation system. The water temperature in both tanks was 13 °C.

### 2.2. Blood Sampling

Blood samples were taken by cardiac puncture into heparinised syringes. Blood was collected immediately after fish capture at the farm in order to avoid transport stress and to minimise the effects of handling. Each blood sample was divided into two parts, one being used for assessment of white blood cell counts, differential cell counts and the phagocyte activity assay, while the other part was centrifuged (1500 rpm, 5 min), and the plasma obtained was frozen at −20 °C until used for determination of complement activity and total immunoglobulin.

After blood collection, the fish were euthanised by stunning with a blow to the head followed by rapid dislocation of the spinal cord. Samples of skin mucus were collected by scraping with a blunt scalpel blade and frozen at −20 °C until used for the lysozyme assay. The fish were then transported to the laboratory and subjected to dissection and parasitological examination.

### 2.3. Pathological and Parasitological Examination; Sampling for Histopathology

All fish were inspected for the presence of external lesions and macroscopically visible ectoparasites, with native skin and gill scrapings examined microscopically. Subsequently, the fish were dissected and examined for the presence of endoparasites and pathological changes to the internal organs. In fish from Sampling B, samples of gill, heart, hepatopancreas, caudal kidney, spleen, and intestine were collected for histopathological examination. Samples were fixed in 10% buffered formalin; dehydrated in a graded ethanol series; embedded in paraffin wax; and 5 μm were sections cut, mounted, and stained with hematoxylin/eosin for histological examination. At least two sections were examined from each tissue sample (four from the gills and two to four from other tissues). Histopathological examination was completed without the knowledge of PCR and clinical status of examined fish. Bacteriological examination has been carried out in two fish from Sampling A. Spleen swabs were cultivated on the blood agar following standard protocol.

### 2.4. Virus Isolation, PCR and Sequence Analysis

Samples of gill, liver, spleen, and kidney tissue were collected from all fish (summarised in Table 1) and examined for the presence of spring viremia of carp virus (carp sprivivirus; SVCV) and other cultivable viruses using standard virological techniques (i.e., virus isolation on EPC and RTG-2 cells). The presence of SVCV, cyprinid herpesvirus 3 (CyHV-3) and CEV was confirmed using nested PCR followed by sequencing [16].

Virus isolation on cell cultures and nested PCR for CyHV-3 and SVCV were not undertaken on Sampling B due to negative results obtained in Sampling A (Table 1).

### 2.5. Haematological and Immunological Parameters

White blood cell counts were assessed according to [21], blood smears were stained using the Hemacolor Rapid staining kit (Merck, Darmstadt, Germany). One hundred leukocytes were counted from each smear and classified as neutrophils, lymphocytes and/or monocytes.

Phagocyte activity (i.e., respiratory burst of neutrophils induced by opsonised zymosan (OZP)) was measured by luminol-enhanced chemiluminescence using a Cytation-3 luminometer (BioTek Instruments, Winooski, VT, USA) using the modified method of Kubala et al. [22]. Lysozyme in skin mucus was determined by radial diffusion in agarose gel containing *Micrococcus luteus* (CCM169), as described by Poisot et al. [23]. Total plasma immunoglobulins were determined using zinc sulphate precipitation, according to McEwan et al. [24]. Quantification of immunoglobulins was based on total protein level as determined using a commercially available kit (Bio-Rad Laboratories, Hercules, CA, USA) before and after precipitation. Final immunoglobulin concentration (in mg·mL^−1^) was calculated as the difference between total plasma protein and proteins present in the supernatant after precipitation and centrifugation. Total complement activity (all activation pathways) was measured using a bioluminescent strain of *Escherichia coli* K12 (pEGFPluxABCDEamp), with the bioluminescence of *E. coli* cells measured using an LM01-T luminometer (Immunotech, Prostejov, Czech Republic) being positively correlated with their viability [25]. The relative measure of complement activity was estimated by computing the difference between the maximal time of measurement (equal to 240 min) and the time necessary (in min) to kill 50% of *E. coli* by the complement (see Buchtíková et al. [26] for further information on this assay).

### 2.6. Statistical Analysis

Normal distribution of variables was tested using the Kolmogorov–Smirnov and Shapiro–Wilk tests. Normally distributed parameters were tested using One-Way ANOVA and post hoc Tukey HSD test for unequal sample size (*Spjøtvoll-Stoline)*. Non-normally distributed parameters were tested using Kruskal–Wallis ANOVA and pairwise comparison. All analyses were performed in Statistica v.13.2.

## 3. Results

### 3.1. Virus Isolation, PCR Analysis and Sequencing

All samples from sampling A provided negative results for presence of CyHV-3 and SVCV over both rounds of nested PCR, with no cytopathic effect on EPC and RTG-2 cells. Analysis for presence of CEV using nested PCR provided PCR products of 528 bp in the first round and 478 bp in the second round from samples of gill tissue (Table 2).

Five of ten gill tissue samples from affected fish in sampling B were positive for the presence of CEV in both rounds of nested PCR, while all pooled organ samples (kidney, liver, and spleen) of affected fish were negative for CEV in both rounds of nested PCR. No PCR products were obtained from pooled gill tissue samples of control fish (Table 2).

**Figure 1:**
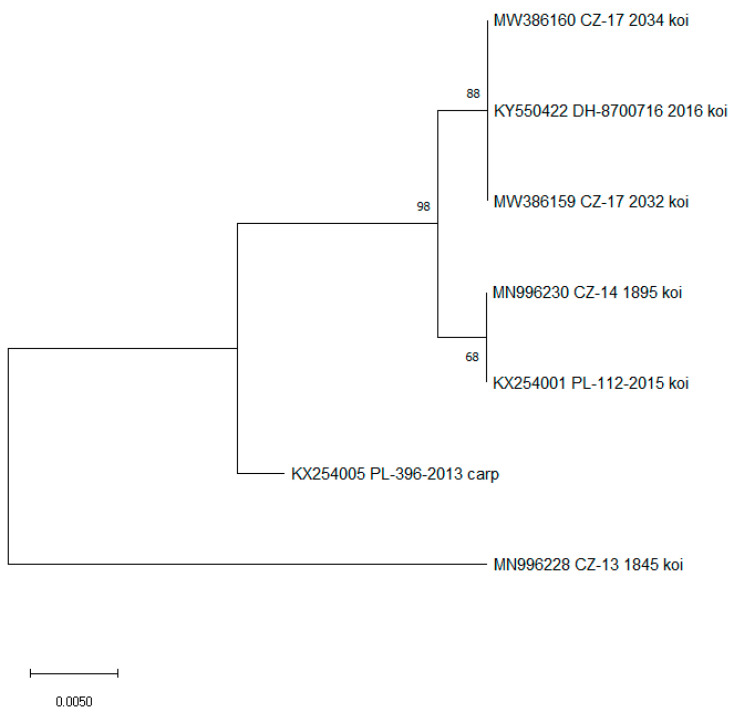
Phylogenetic analysis of the 357-bp nucleotide sequence encoding the CEV P4a core protein. This analysis presents the sequences obtained in the present study from the affected koi in the Czech Republic (marked) and other CEV sequences of common and koi carp obtained from GenBank; Germany (DH), Hungary (HU), Poland (PL), Slovakia (SVK), and Czech Republic (CZ). A Maximum Likelihood Tree was constructed using a Jukes-Cantor model, and the robustness of the tree was tested using 1000 bootstrap replicates. The branch length is proportional to the number of substitutions per site.

Sequencing of positive samples from Sampling A and Sampling B showed 100% nucleotide sequence identity. The sequences have been listed in GeneBank under accession numbers MW386159 and MW386160. Phylogenetic analysis of the 357-bp nucleotide sequence encoding the CEV 4a gene showed that the virus detected in our samples belonged to CEV genogroup IIa, which is mainly detected in koi carp. The virus shared the same nucleotide sequence as a CEV isolate detected from koi carp in Germany in 2016 (accession number KY550422, Figure 1).

### 3.2. Gross and Microscopic Pathology, Results of Parasitological and Bacteriological Examination

Pathoanatomical findings in fish displaying clinical signs are summarized in Table 1. No pathological changes were found in control fish.

**Figure 2:**
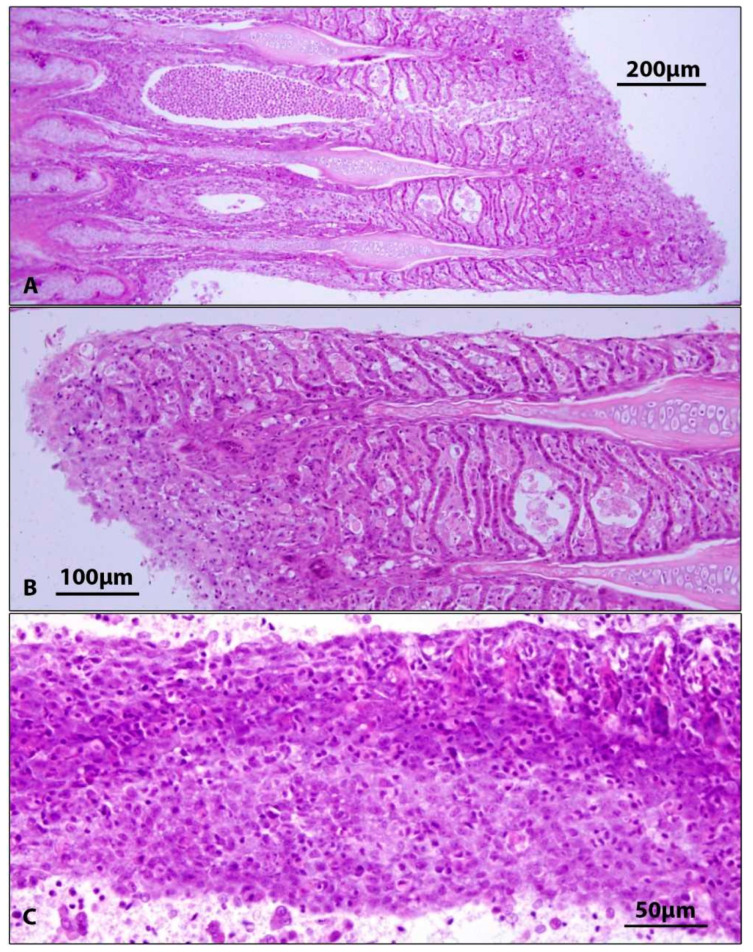
Gills of CEV+ fish. (**A**,**B**). Fusion of gill secondary lamellae and cyst-like formation with cellular debris. (**C**). Focus of gill filament alterative inflammation. H&E.

None of the fish had macroscopic gill lesions, but all diseased fish (both CEV+ and CEV−) had various stages of gill damage, ranging from epithelial hyperplasia and fusion of secondary lamellae to desquamation of gill epithelium with an absence of secondary lamellae. Cyst-like spherical formations were between adjacent secondary lamellae (Figure 2, Table 3).

**Figure 3:**
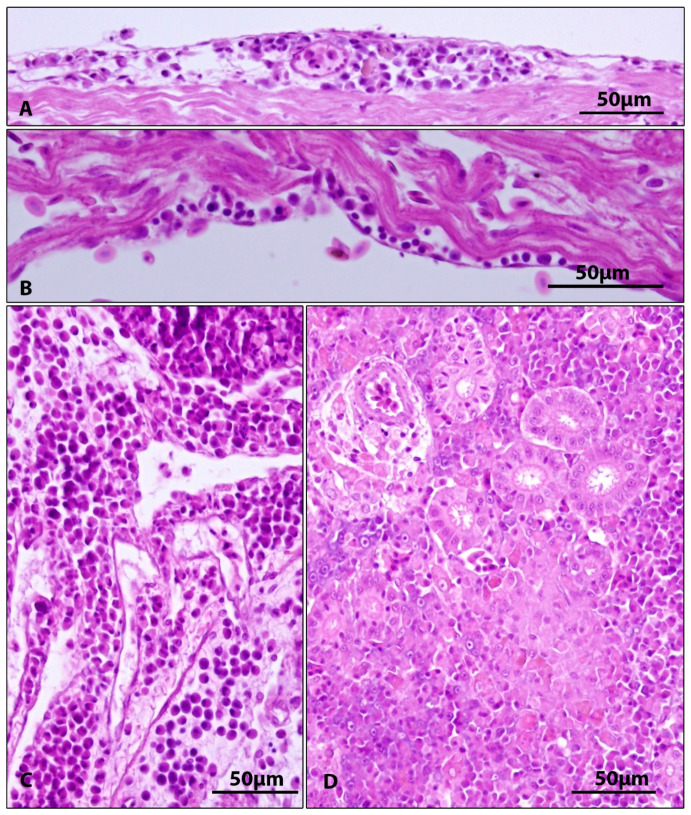
CEV+ fish. (**A**,**B**). Subepicardial (**A**) and subendocardial (**B**) inflammatory infiltration. (**C**). Inflammatory infiltration of connective tissue surrounding ventral lobe of caudal kidney. (**D**). Proliferation of kidney interstitium, necrotic focus with previously proliferated aggregate of macrophages. H&E.

**Figure 4:**
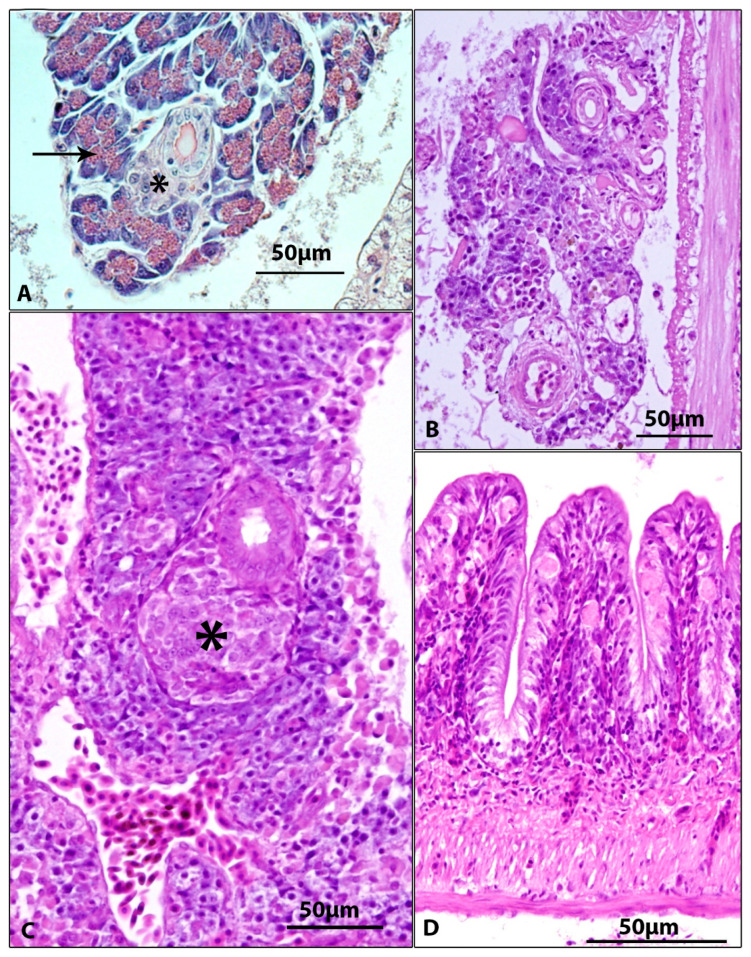
CEV + fish. (**A**). Hepatopancreas of control fish. Liver tissue, endocrine pancreas (*) and exocrine pancreas with zymogen granules (↑). (**B**,**C**). Absence of exocrine pancreas and atrophy of endocrine pancreas (*). (**D**). Spheric formations (most probably of macrophage origin) in the epithelial lining of the intestine. H&E.

Histopathological findings in other organs included subepicardial and subendocardial infiltrate (Figure 3A,B, Table 3), inflammatory infiltration of connective tissue between internal organs (Figure 3C, Table 3), and proliferation of kidney interstitium, with macrophage aggregation (Figure 3D). The exocrine and endocrine pancreas tissues were atrophic in all diseased fish (Figure 4B,C), with loss of zymogen granules in exocrine pancreas (Table 3). In the intestine, subepithelial infiltrate and spherical formations (probably of macrophage origin) were present in the epithelium (Figure 4D, Table 3).

Only a single *Dactylogyrus* sp. was present on the gills of one of the diseased fish.

Systemic bacterial infection was confirmed in both fish from Sampling A (*Aeromonas bestiarum* in one fish and *Aeromonas hydrophila* in the other one).

### 3.3. Leukocyte Counts, Differential Cell Counts, and Immunological Parameters

As five fish showing clinical signs of the disease proved CEV-negative by nested PCR, results of haematological, histopathological, and immunological examination are presented separately for CEV+ fish, CEV− fish, and control fish from another tank (Table 1, Table 3, and Table 4). Significantly decreased lymphocytes compared to controls were found in all diseased fish (both CEV+ and CEV−). Lymphopenia was accompanied by marked neutrophilia in CEV+ fish, which was, however, insignificant due to high variation among measured values. White blood cell counts were significantly decreased in CEV-fish compared to controls (Table 4).

**Figure 5:**
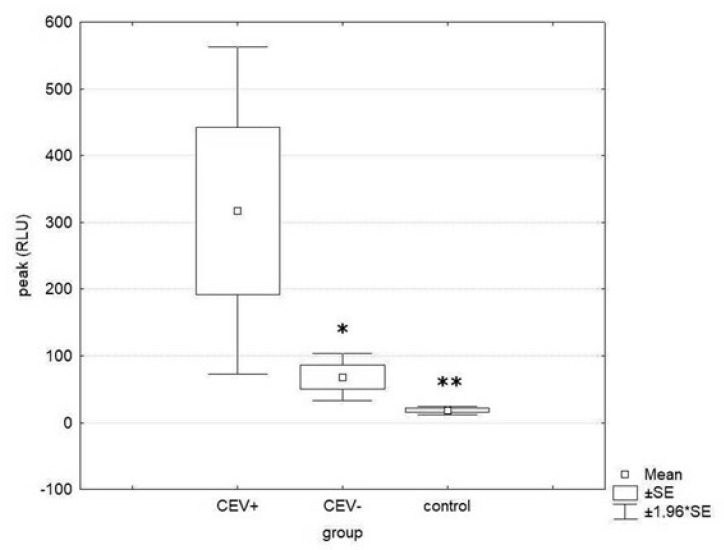
Phagocyte activity in CEV-positive, CEV-negative, and clinically healthy CEV-negative control fish from another tank. The data represent peaks of measured kinetic curves expressed as relative light units (RLU). Statistically significant differences are marked with asterisks (* *p* < 0.05; ** *p* < 0.01).

Phagocyte activity, expressed as a peak of measured kinetic curves, was significantly higher in CEV+ fish than in controls (*p* = 0.0083) and CEV− fish (*p* = 0.0269) (Figure 5). When phagocyte activity was expressed as chemiluminescence signal per 1000 phagocytes, allowing us to evaluate it independently of phagocyte count, no statistically significant differences were found among groups (Table 4). Other immunological parameters measured in this study (complement activity, lysozyme activity, and level of total immunoglobulins) did not show significant differences among groups.

## 4. Discussion

In the present study, we show marked disturbances in white blood cell counts in diseased fish. Lymphocyte counts significantly decreased in all diseased fish (both CEV+ and CEV−). However, in CEV+ fish, lymphocyte depletion was offset by the strong increase in neutrophils, and consequently, total leukocyte counts in CEV+ group were similar to values found in healthy controls. Decrease in total white blood cell counts with neutrophilia and lymphopenia was reported by Lewisch et al. [11] in CEV-affected koi. Additionally, the work of Adamek et al. [27] performed under controlled laboratory conditions shows decreased white blood cell counts and lymphocyte depletion accompanied by granulocytosis in CEV-infected koi. The systemic decrease in lymphocyte counts is associated with a variety of viral infections in both animals and humans (reviewed by Guo et al. [28]. Lymphocytes are a crucial part of adaptive immunity and perform an important role in the control of viral infections. In various virus infections, the degree of lymphopenia was shown to be associated with disease severity [28]. It also seems that lymphopenia is associated with an increased risk of infections of various aetiology and with an increased risk of infection-related death [29]. In CEVD, lymphopenia may be one of the factors supporting development of intercurrent infections, which are often diagnosed in CEV-affected fish [20]. A decrease in the lymphocyte count is also a common finding during stress not only in fish but also in other vertebrates [30], and it is considered a nonspecific response independent of the type of stress trigger [31]. On the contrary, neutrophils are usually increased during the stress response [30]. Results of the present study show only overall lymphopenia without indicating which lymphocyte subpopulations were depleted. The work of Adamek et al. [27] reports the downregulation of CD4, a surface protein of T-helper lymphocytes and the downregulation of T cell receptor a2 in CEV-infected koi.

There was a strong increase in the respiratory burst of phagocytes in CEV+ fish. Phagocytosis associated with respiratory burst is a highly efficient defence microbicide mechanism regarded as the first line of defence against invading microorganisms [32]. Respiratory burst intensity is dependent on NADPH oxidase activity, which can be enhanced by proinflammatory cytokines, lipopolysaccharides and/or other agents [33]. When phagocyte activity in our study was expressed as chemiluminescence signal per 1000 phagocytes, allowing us to evaluate phagocyte activity independently of the phagocyte count, no statistically significant difference was found between CEV+, CEV−, and control fish. This suggests that the increase in respiratory burst in diseased fish was caused by the increase in phagocyte counts and not by an increase in metabolic activity of individual phagocytic cells. From the perspective of the whole organism, however, phagocytic activity was several times higher in diseased fish than in controls, which suggests enhancement of this part of nonspecific immune response. Other immunological parameters focused on in this study (i.e., complement activity, lysozyme activity, and level of total immunoglobulins) were not significantly altered in diseased fish. The work of Adamek et al. [27] describes a decrease in abundance of IgM transcripts in the gills and kidneys of CEV-affected koi. In the present study, plasma immunoglobulins decreased in CEV+ fish compared to CEV− and controls, but without statistical significance. It should be noted, however, that the water temperature in the tank was 13 °C, which does not allow intensive immunoglobulin synthesis in carp [34,35] and, hence, the development of marked differences among diseased and healthy fish. As such, further research targeted on a wider array of immunological parameters will be needed to evaluate the immune status of CEV-diseased fish in a greater detail.

No macroscopic changes on gills were found in diseased fish despite the fish displaying marked lethargic behaviour. This is in accordance with the findings of Adamek et al. [36], who reported a mismatch between the severity of clinical symptoms and pathological findings on the gills. Histopathological changes found on gills of diseased fish (hyperplasia of gill epithelium, fusion of adjacent secondary lamellae, desquamation of gill epithelium) corresponded to previous reports [1,6,11,18,20,37]). Although histopathological changes were similar in all diseased fish, only five of ten fish proved CEV-positive on PCR. This can be explained by a different stage of the disease and potential differences in viral loads. Gill damage and clinical signs might persist even after CEV loads have already declined below the detection limit of the nested PCR. Although the gills are the main target organ of CEV [36], other organs (heart, kidney, skin, intestine, and pancreatic tissue) also showed histopathological changes. Microscopic changes on internal organs of CEV-affected fish were described by several authors, more commonly in koi [6,11,38] than in common carp [39]. Previous studies described kidney swelling, degeneration, and necrosis of tubular epithelial cells, inflammatory infiltration of renal interstitium [6,11], congestion of renal interstitium [39], necrotic lesions in spleen and hepatopancreas, myocarditis, and diffuse enteritis [11]. Our findings (inflammatory infiltrate in connective tissue between internal organs, proliferation of kidney interstitium with macrophage infiltrates, and subepithelial inflammatory infiltrate in the intestine) largely correspond with above mentioned results. In addition, the present study, for the first time, shows extensive atrophy of the pancreatic tissue (both exocrine and endocrine) with missing zymogen granules in acinar cells of exocrine pancreas. As indicated above, histopathological changes in internal organs are frequently found in CEV-diseased fish. However, it has been found that virus loads in internal organs are very low compared to virus loads in gills. For example, Adamek et al. [27,36] found that virus loads in internal organs (kidney, spleen hepatopancreas, gonads, gut, and brain) of diseased carp were 3–4 orders of magnitude lower than in gills. In the present work, we did not detect CEV in pooled samples of organs at all. Histopathological changes caused by virus infections may be a result of direct lytic activity of the virus on target tissue but also a result of massive activation of immune system (leukocyte infiltration to tissues and activation of resident immune cells) triggered by viremia [40] and this tissue damage may persist even after viraemic phase of the disease. This may explain the presence of histopathological changes in organs in which virus has not been detected. Another issue is frequent occurrence of intercurrent infections in CEV-affected fish from field cases [5,9,11,12,15]. Damaging effect of CEV on internal organs should be verified by further research under controlled experimental conditions.

The owner reports that fish were transported from one facility to another prior to the first occurrence of clinical symptoms. Handling and transport are important triggers of stress, which is known to be one of the major factors supporting disease development [41]. Stress-related reactivation of persistent CEV infection was suggested by Adamek et al. [42]. Similar to the present study, Lewisch et al. [11] and Pikula et al. [43] reported CEVD cases in which restocking probably performed a significant role in the disease outbreak.

In conclusion, the present study shows changes in immune-related cell counts, reflected by an increased intensity of respiratory burst of phagocytes and newly documented histopathological changes in the pancreatic tissue of diseased koi.

## Figures and Tables

**Table 1:** Results of pathoanatomical, parasitological, and bacteriological examination. n. d.—not done.

Sampling Date	Water Temperature	Fish	Pathoanatomical Examination	Parasitological Examination	Bacteriological Examination
4 April 2017 (Sampling A)	12 °C (Tank 1)	Koi carp, diseased (*n* = 2)	Poor condition (2/2) Skin lesions with loss of scales (2/2) Enophthalmia (2/2)	No parasites	*Aeromonas bestiarum* (1/2), *Aeromonas hydrophila* (1/2)
17 April 2017 (Sampling B)	13 °C (Tank 1)	Koi carp, diseased, CEV+ (*n* = 5)	Enophthalmia, poor condition (3/5)	No parasites	n. d.
		Koi carp diseased, CEV− (*n* = 5)	Haemorrhagic fluid in body cavity (1/5) Bristled scales, poor condition (1/5)	*Dactylogyrus* sp., 1× (1/5)	
	13 °C (Tank 2)	Koi carp, control (*n* = 10)	No gross pathology	No parasites	

**Table 2:** Results of virological examination and nested PCR. SVCV—spring viraemia carp virus, CyHV-3—cyprinid herpesvirus type 3, CEV—carp edema virus, k = kidney, l = liver, s = spleen. + = positive result, − = negative result, n. d. = not done.

Sampling Date	Fish	Sample Type	SVCV (Cell Culture)	SVCV (Nested PCR)	CyHV-3 (Nested PCR)	CEV (Nested PCR) 1st Round/ 2nd Round
4 April 2017 (Sampling A)	Koi carp, diseased	k, l, s (pooled from 2 fish)	−	−	−	−/−
		gills (pooled from 2 fish)	−	−	−	+/+
17 April 2017 (Sampling B)	Koi carp, diseased	k, l, s (pooled from each fish), *n* = 10	−	−	−	−/−
		gills, *n* = 10	−	−	−	+/+ (5 fish of 10)
	Koi carp, control	gills (pooled from 10 fish)	−	−	−	−/−

**Table 3:** Results of histopathological examination, 17 April 2017 (sampling B).

	Koi Carp, Diseased, CEV+ (*n* = 5)	Koi Carp Diseased, CEV− (*n* = 5)	Figure
Gills	Epithelial hyperplasia, fusion of secondary lamellae, cystoid formations between gill lamellae (5/5)	Epithelial hyperplasia, fusion of secondary lamellae, cystoid formations between gill lamellae (5/5)	Figure 2
Pancreas	Absence of pancreatic tissue (2/5) atrophy of pancreas with loss of zymogen granules in exocrine pancreas (3/5)	Absence of pancreatic tissue (1/5) atrophy of pancreas with loss of zymogen granules in exocrine pancreas (4/5)	Figure 4B,C
Intestine	Subepithelial infiltrate and presence of spherical formations (probably of macrophage origin) (5/5)	Subepithelial infiltrate and presence of spherical formations (probably of macrophage origin) (3/5)	Figure 4D
Heart	Subepicardial and subendocardial infiltrates (2/5)	Subepicardial and subendocardial infiltrates (2/5)	Figure 3A,B
Kidney	Proliferation of kidney interstitium, with macrophage aggregation (5/5)	Proliferation of kidney interstitium, with macrophage aggregation (3/5)	Figure 3D
Body cavity	Inflammatory infiltration of connective tissue among internal organs (1/5)	Not detected	Figure 3C

**Table 4:** White blood cell counts, differential cell counts, and immunological parameters in CEV-positive fish (CEV+), CEV-negative fish from the same tank (CEV−), and clinically healthy CEV-negative control fish from another tank. Data represent means ± standard deviations. Statistically significant differences between groups are marked with different letters.

	Control	Diseased CEV+	Diseased CEV−
Leukocytes (10^9^·L^−1^)	44.0 ± 33.19 a	42.8 ± 41.88 ab	19.2 ± 11.58 b
Lymphocytes (%)	91.8 ± 9.67 a	56.0 ± 37.86 b	77.6 ± 6.02 b
Neutrophils (%)	7.1 ± 8.96	43.6 ± 37.25	19.2 ± 5.071
Monocytes (%)	1.1 ± 1.29 a	0.40 ± 0.89 b	3.20 ± 2.59 ab
Phagocytes (%)	8.2 ± 9.67	44.0 ± 37.86	22.4 ± 6.02
Lymphocytes (10^9^·L^−1^)	39.85 ± 29.72 a	12.79 ± 29 b	14.97 ± 9.51 b
Neutrophils (10^9^·L^−1^)	3.78 ± 7.6	29.56 ± 42.25	3.7 ± 2.24
Monocytes (10^9^·L^−1^)	0.37 ± 0.36 a	0.45 ±1.01 b	0.53 ± 0.32 ab
Phagocytes (10^9^·L^−1^)	4.15 ± 7.86	30.0 ± 43.2	4.23 ± 2.41
Phagocytic activity (peak)	18.3 ± 9.5 a	317.5 ± 279.7 b	67.8 ± 40.4 a
Phagocytic activity (peak/1000 phagocytes)	1.59 ± 1.72	1.73 ± 1.48	1.05 ± 0.90
Lysozyme (mg·mL^−1^)	11.02 ± 4.23	14.62 ± 4.93	11.76 ± 5.75
Complement activity (min)	46.8 ±27.5	49.4 ± 23.7	34.3 ± 24.3
Immunoglobulins (mg·mL^−1^)	12.3 ± 3.47	9.01 ± 2.40	11.07 ± 7.74

## Data Availability

All data analysed in the present study are available from the corresponding author upon request.
